# Isoliquiritigenin induces apoptosis and autophagy and inhibits endometrial cancer growth in mice

**DOI:** 10.18632/oncotarget.12369

**Published:** 2016-09-30

**Authors:** Chi-Hao Wu, Hsin-Yuan Chen, Chia-Woei Wang, Tzong-Ming Shieh, Tsui-Chin Huang, Li-Chun Lin, Kai-Lee Wang, Shih-Min Hsia

**Affiliations:** ^1^ School of Nutrition and Health Sciences, College of Nutrition, Taipei Medical University, Taipei, Taiwan; ^2^ Graduate Institute of Clinical Medicine, College of Medicine, Taipei Medical University, Taipei, Taiwan; ^3^ Department of Obstetrics and Gynecology, School of Medicine, College of Medicine, Taipei Medical University, Taipei, Taiwan; ^4^ Department of Obstetrics and Gynecology, Taipei Medical University Hospital, Taipei, Taiwan; ^5^ Department of Dental Hygiene, College of Health Care, China Medical University, Taichung, Taiwan; ^6^ PhD Program for Cancer Biology and Drug Discovery, College of Medical Science and Technology, Taipei Medical University and Academia Sinica, Taipei, Taiwan

**Keywords:** endometrial cancer, isoliquiritigenin, apoptosis, autophagy

## Abstract

Endometrial cancer is the most common cancer in women, typically with onset after menopause. Isoliquiritigenin (ISL), a licorice flavonoid, was previously shown to have anti-oxidant, anti-inflammatory, and tumor suppression effects. In this study, we investigated the anti-tumor effect of ISL on human endometrial cancer both *in vitro* and *in vivo*. We used telomerase-immortalized human endometrial stromal cells (T-HESCs) and human endometrial cancer cell lines (Ishikawa, HEC-1A, and RL95-2 cells) as targets. The effects of ISL on cell proliferation, cell cycle regulation, and apoptosis or autophagy-related protein expression were examined. In addition, we conducted *in vivo* experiments to confirm the inhibitory effects of ISL on cancer cells. ISL significantly inhibited the viability of cancer cells in a dose- and time-dependent manner but with little toxicity on normal cells. In addition, flow cytometry analysis indicated that ISL induced sub-G1 or G2/M phase arrest. ISL treatment activated the extracellular signal regulated kinase signaling pathway to enhance the protein expression of caspase-7/LC3BII associated with apoptosis/autophagy. Furthermore, ISL suppressed xenograft tumor growth *in vivo*. Taken together, these findings suggest that ISL may induce apoptosis, autophagy, and cell growth inhibition, indicating its potential as a therapeutic agent for human endometrial cancer.

## INTRODUCTION

Endometrial cancer is the most common malignant tumor of the female reproductive tract, with onset typically after menopause. In Taiwan, the incidence rate of this cancer from 2006 to 2010 was 37.52 per 100,000 women per year in women aged between 55 and 59 years [1]. Moreover, the incidence rate in younger women has been increasing, with up to 14% of cases diagnosed in premenopausal women. Women younger than 40 years account for 4% of endometrial cancer cases [2–3]. Endometrial cancers are usually treated with surgery, radiation, hormones, and chemotherapy, depending on the stage of disease. However, for endometrial precancer patients who desire to maintain their fertility, hysterectomy may not be an ideal management choice.

Recently, the widespread use of herbal therapies and traditional Chinese medicine in western countries has been rising owing to their demonstrated effectiveness in cancer therapy [4]. Isoliquiritigenin (ISL), a natural flavonoid isolated from the root of licorice (*Glycyrrhiza uralensis*), has a chalcone structure (4, 20, 40-trihydroxychalcone) [5]. ISL has various biological properties, including anti-inflammatory, anti-oxidant, anti-platelet aggregation, vasorelaxant, and estrogenic effects [6–10]. It is also a potent mitotic inhibitor and an inducer of apoptosis, suggesting it may have significant anti-tumor activities [11–12]. Furthermore, studies have shown that ISL can induce autophagy in many cancer cell lines [13].

A recent study demonstrated that dietary agents that prevent tumorigenesis by disrupting tumor initiation, proliferation, and propagation via various cell death pathways, such as apoptosis, necrosis, and autophagy [14]. Apoptosis is type-I programmed cell death and is characterized by membrane blebbing, cell shrinkage, cell membrane phosphatidylserine valgus, nuclear fragmentation, chromatin condensation, mitochondrial membrane potential depolarization, and chromosomal DNA fragmentation. Hydrolytic enzymes perturb the cytoplasmic architecture, leading to the generation of apoptotic bodies and finally to the induction of apoptosis [15]. Autophagy has dual roles in cancer, acting as both a tumor suppressor by the selective degradation of proteins and damaged organelles and as a promoter of cell survival by increasing tumor growth. Autophagic cell death, or type-II programmed cell death, is characterized by the accumulation of multilamellar vesicles that engulf the cytoplasm and organelles [16–17]. There are three different relationships between autophagy and apoptosis: autophagy may be indispensable for the occurrence of apoptosis, autophagy may antagonize apoptosis, and apoptosis and autophagy may occur independently of each other. Inhibition of autophagic activity in cells may increase death signals by switching from autophagic type II programmed cell death to apoptotic type I cell death [18]. In the present study, we investigated the anti-tumor effect of ISL on human endometrial cancer *in vitro* and *in vivo*. Furthermore, we examined the relationship between autophagy and apoptosis.

## RESULTS

### ISL inhibits proliferation of cancer cells

**Figure 1:**
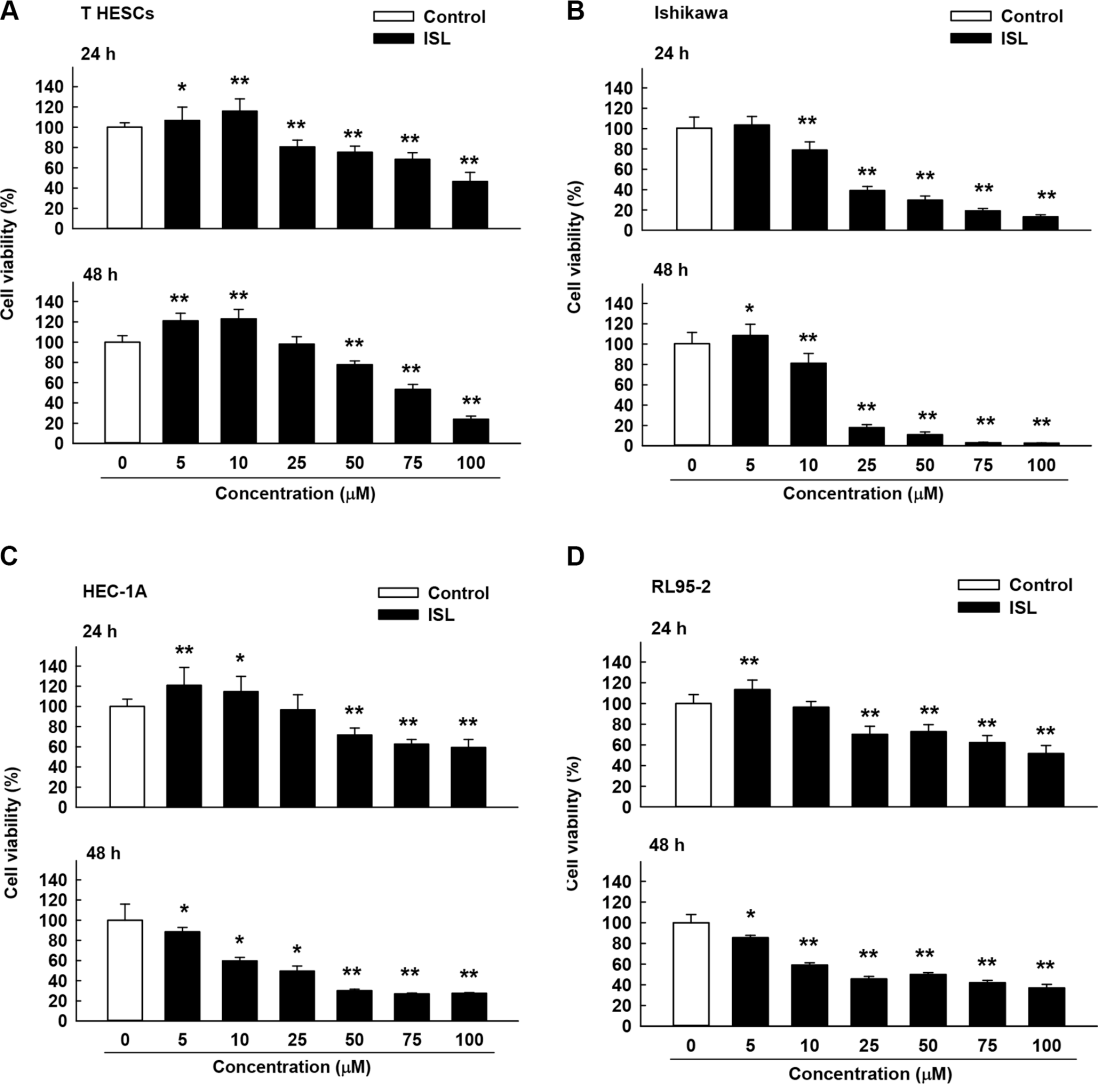
Cytotoxicity of isoliquiritigenin (ISL) in human endometrial cancer cell lines T HESCs and Ishikawa, HEC-1A, and RL95-2 cells were treated with vehicle or ISL (5 to 100 μM) for 24–48 h. Cell proliferation was measured by MTS assay. Results are expressed as means ± standard deviation (SD) from three independent experiments. *p < 0.05 and **p < 0.001 compared with control.

**Figure 2:**
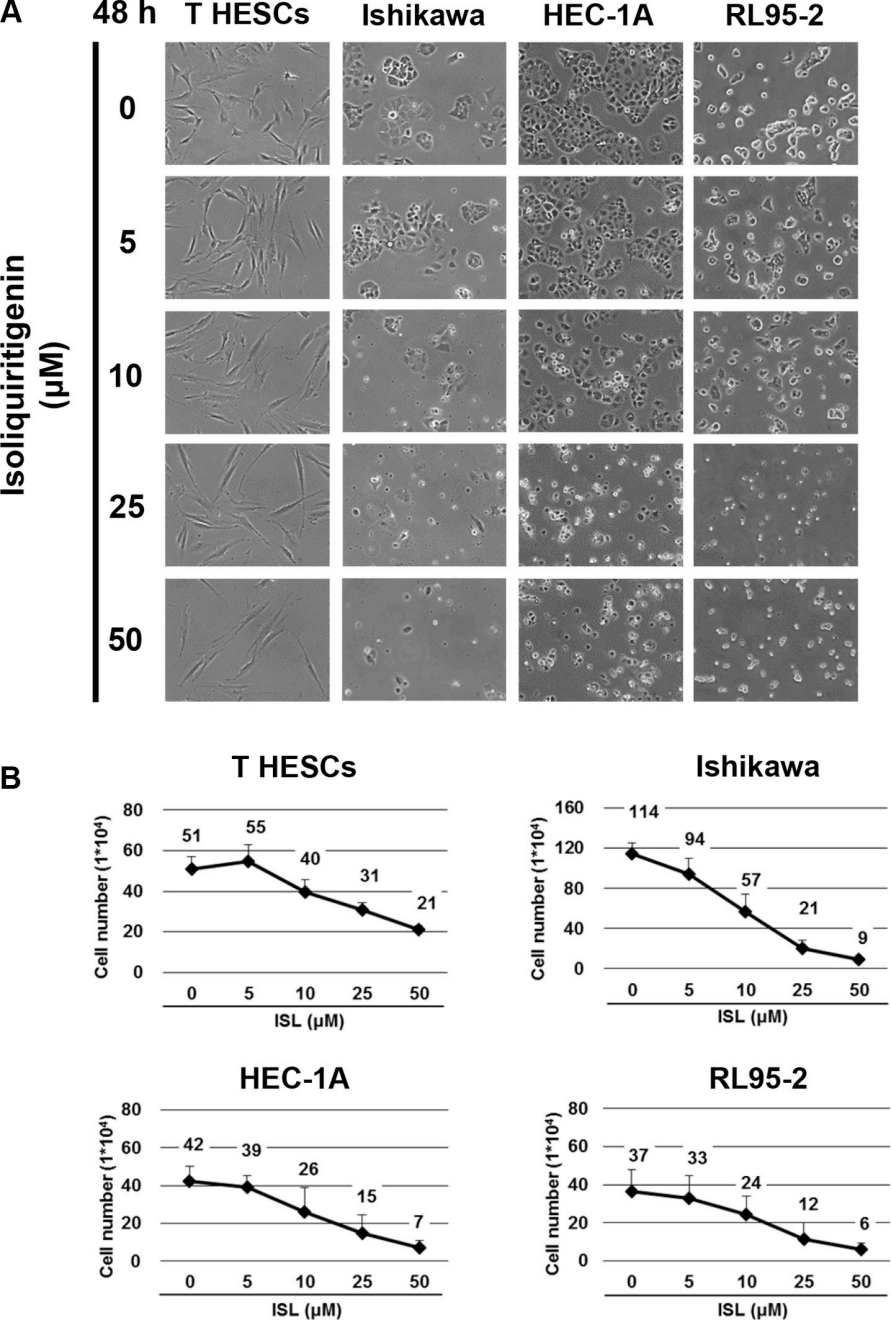
The anti-proliferative effect of ISL on human endometrial cancer cell lines Cells were plated in 6-well plates at a density of 5 × 10^4^ cells/well in media containing 10% fetal bovine serum (FBS) and allowed to adhere. (**A**) The morphology of T HESCs and Ishikawa, HEC-1A, and RL95-2 cells after treatment with vehicle or 5, 10, 25, and 50 μΜ of ISL in media containing 1% FBS for 48 h. (**B**) The number of viable cells after treatment with vehicle or ISL (5, 10, 25, 50 μΜ) in media containing 1% FBS for 48 h. Cell viability was determined by the trypan blue exclusion test. Results are expressed as means ± SD from three independent experiments.

The effect of ISL on the viability of endometrial cancer cell lines (Ishikawa, HEC-1A, and RL95-2) was examined by treating cells with vehicle control and increasing concentrations of ISL (5, 10, 25, 50, 75, and 100 μM). Normal endometrial cells, T-HESCs, were used as a control. Cell viability was measured by MTS assay at 24 and 48 h. ISL treatment decreased the growth of cancer cell lines in a dose- and time-dependent manner (Figure 1B, 1C and 1D); however, the viability of T-HESCs showed slight changes up to an ISL concentration of 59 μM (Figure 1A). These results indicated that ISL inhibited the growth of cancer cells but has little effect on normal cells at concentrations below 27 μM. Similarly, as shown in Figure 2B, ISL inhibited the growth of endometrial cancer cell lines (Ishikawa, HEC- 1A and RL95-2) and promoted the formation of rounded floating cells (Figure 2A).

### ISL induces DNA damage, cell cycle arrest, and apoptotic cell death in endometrial cancer cells

**Figure 3:**
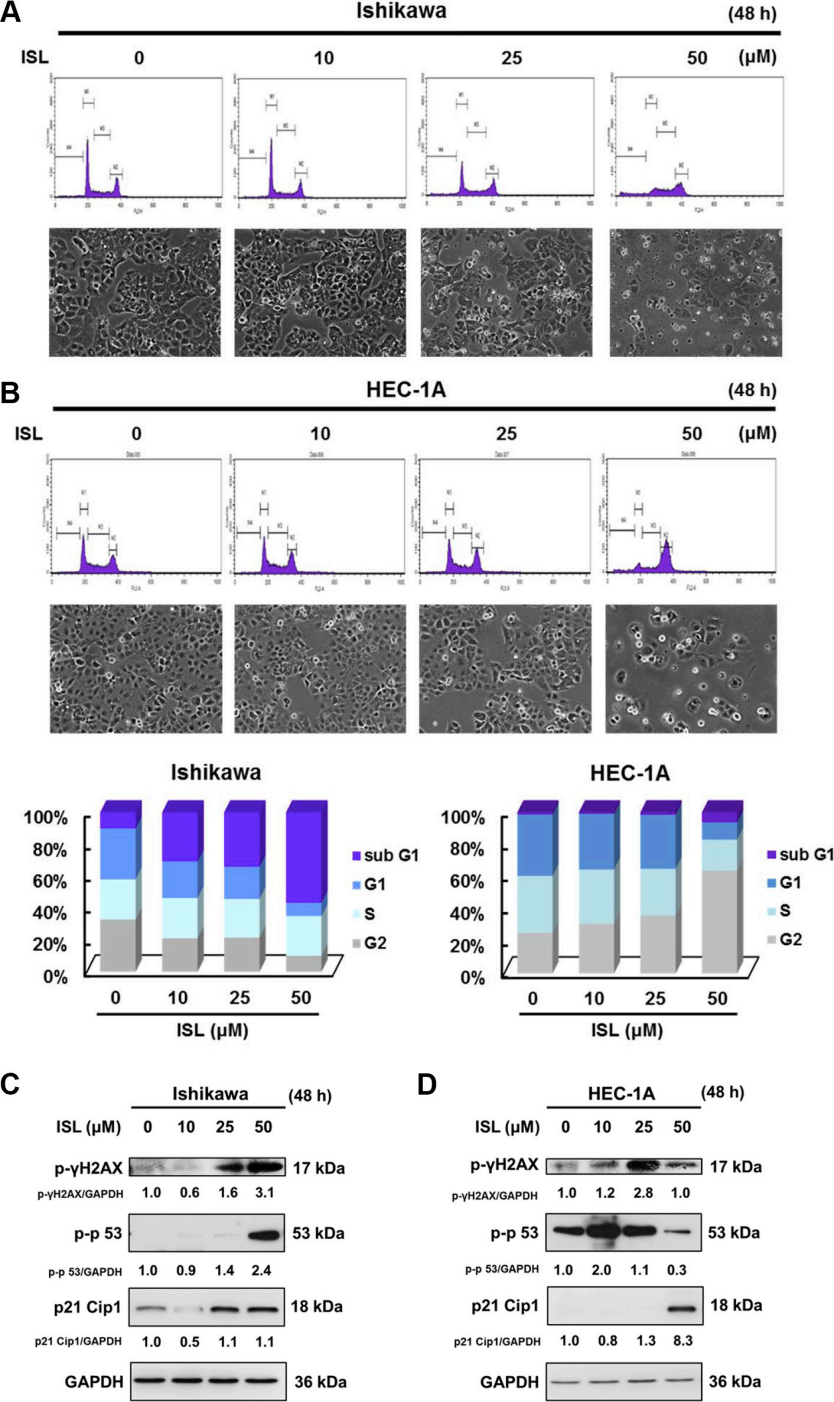
ISL induces cell cycle arrest in human endometrial cancer cells Cells were plated in 100 mm dishes at a density of 1 × 10^6^ cells/plate in media supplemented with 10% FBS and allowed to adhere. The morphology of cells after treatment with vehicle or ISL (10, 25, 50 μΜ) in media containing 1% FBS for 48 h. (**A**) Ishikawa and (**B**) HEC-1A cells were stained with propidium iodide (PI), and cell cycle distribution was analyzed by flow cytometry. The vertical axis represents the number of cells, and the horizontal axis represents the intensity of PI staining. The cell cycle distribution is shown as a bar graph. The vertical numbers represent the cell population percentage in cell cycles S, G2, and G1phase, and the horizontal numbers represent the concentration of ISL. (**C**) Ishikawa and (**D**) HEC-1A cell lysates were separated by sodium dodecyl sulfate polyacrylamide gel electrophoresis (SDS-PAGE) and analyzed by western blot with the indicated antibodies. Glyceraldehyde 3-phosphate dehydrogenase (GAPDH) was used as a loading control. The values of the band intensity represent the densitometric estimation of each band normalized to GAPDH.

To examine whether the cytotoxic property of ISL is associated with cell cycle arrest, we examined the effect of ISL on cell cycle progression. We found that ISL alone dose-dependently arrested the cell cycle of Ishikawa cells at the sub G1 checkpoint (Figure 3A) and arrested HEC- 1A cells at the G2/M checkpoint (Figure 3B).

To elucidate the mechanism underlying the induction of cell cycle arrest by ISL in endometrial cancer cells, the expression of proteins that regulate cell cycle progression was analyzed by western blot. As shown in Figure 3, ISL treatment activated the DNA damage marker γ-H2AX and significantly elevated the phosphorylation of p53 (a tumor suppressor protein) and p21 Cip1 (a downstream target of p53) in Ishikawa (Figure 3C) and HEC-1A cells (Figure 3D). Therefore, ISL induced cytotoxicity, at least in part, by promoting cell cycle arrest.

**Figure 4:**
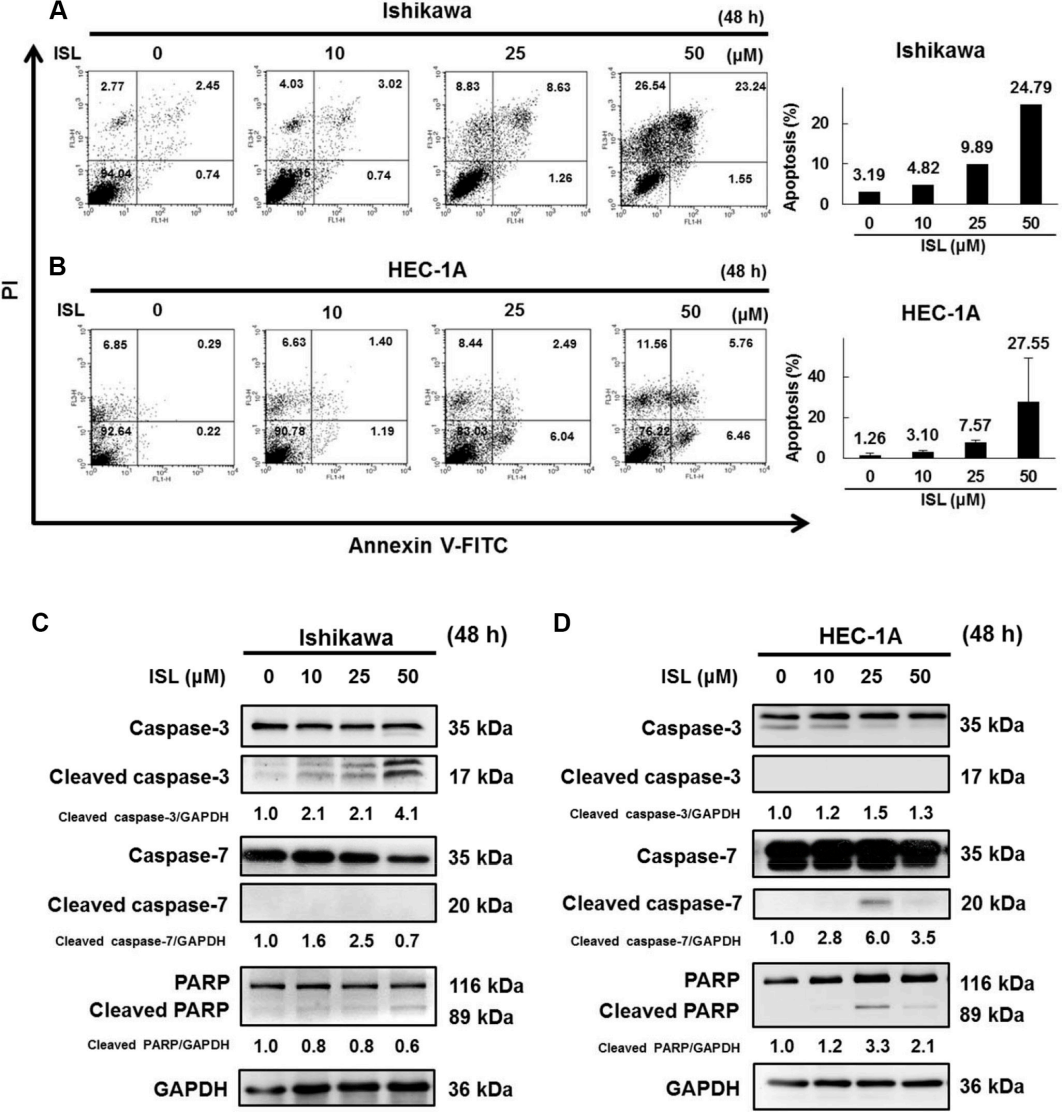
ISL induces apoptosis in human endometrial cancer cells Cells were treated with ISL (0, 10, 25, 50 μM) for 48 h. (**A**) Ishikawa and (**B**) HEC-1A cells were harvested and stained with Annexin V-fluorescein isothiocyanate (FITC) and PI, and cell apoptosis was analyzed using flow cytometry. (**C**) Ishikawa and (**D**) HEC-1A cell lysates were separated by SDS-PAGE and analyzed by western blot with the indicated antibodies. GAPDH was used as a loading control. The values of the band intensity represent the densitometric estimation of each band normalized to GAPDH.

Next, we investigated whether ISL could induce apoptosis in endometrial cancer cells. Flow cytometry was used to examine annexin V-FITC and PI double staining and revealed a significant shift in annexin V-FITC-positive cells after ISL treatment in Ishikawa (Figure 4A) and HEC-1A cells (Figure 4B). In Ishikawa cells, western blot showed that levels of both cleaved caspase-3 and cleaved PARP increased after ISL treatment (Figure 4C), whereas only a small change in caspase-7 level was observed. In HEC-1A cells, the protein expression of cleaved caspase-7 and cleaved PARP was greatly upregulated after ISL (25 μM) treatment (Figure 4D), but there was no change in caspase-3 level. Therefore, ISL treatment activated different pathways to induce apoptotic cell death in Ishikawa and HEC-1A cells.

### ISL triggers autophagy in HEC-1A cells

**Figure 5:**
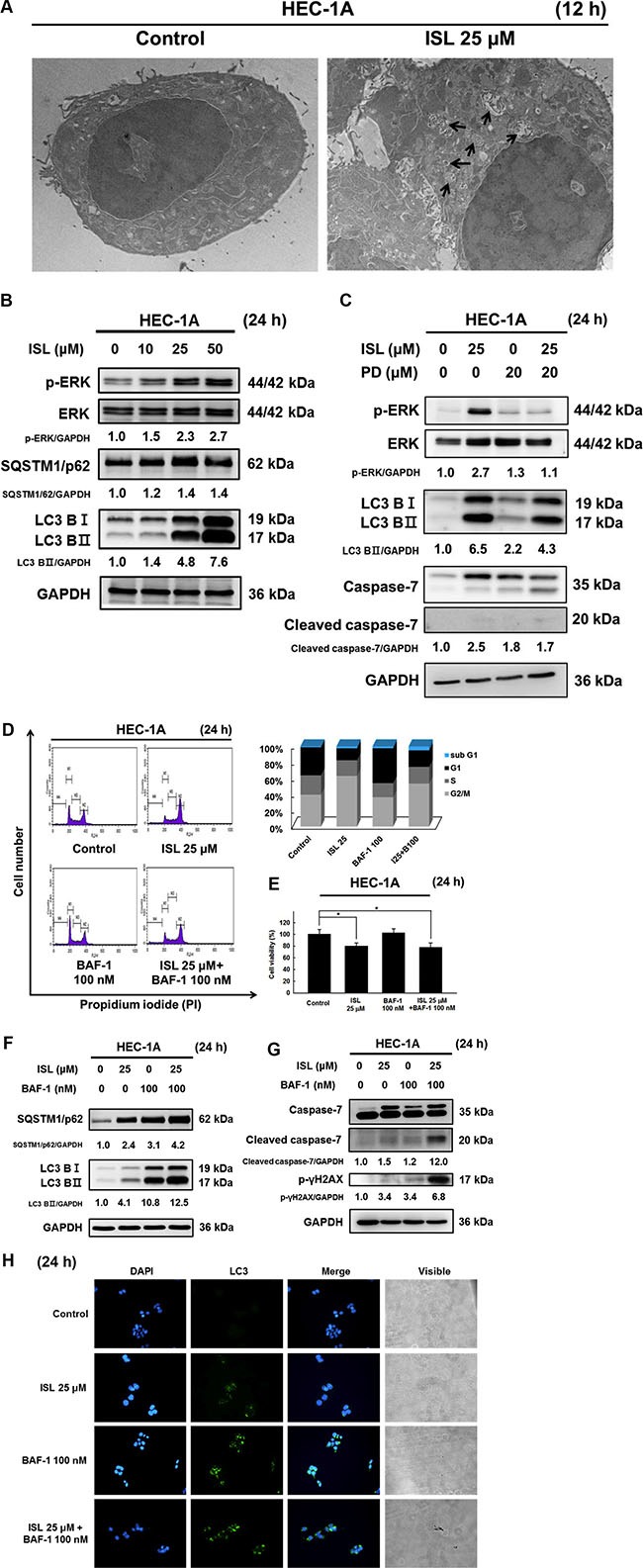
ISL triggered protective autophagy in HEC-1A cells (**A**) Transmission electron microscope (TEM) analysis of tumor sections obtained from control and ISL treated groups. (**B**) HEC-1A cells were treated with ISL (0, 10, 25, 50 μM) for 24 h. (**C**) Treatment with ISL (25 μM) for 24 h with or without MEK inhibitor (PD98059) pretreatment (20 nM, 1 h). (**F**) and (**G**) Treatment with ISL (25 μM) for 24 h with or without BAF pretreatment (100 nM, 4 h). Cell lysates were separated by SDS-PAGE and analyzed by western blot with the indicated antibodies. GAPDH was used as a loading control. The values of the band intensity represent the densitometric estimation of each band normalized to GAPDH. (**D**) Treatment with ISL (25 μM) for 24 h with or without BAF pretreatment (100 nM, 4 h). Cells were stained with PI and cell cycle distribution was analyzed by flow cytometry. (**E**) The number of dead cells was determined using trypan blue. The results are expressed as means ± SD of three independent experiments. **p < 0.001 compared with control. (**H**) HEC-1A cells were cultured in ISL (25 μM) for 24 h with or without BAF pretreatment (100 nM, 4 h), and the intensity of green fluorescence (LC3) was observed, original magnification, ×200.

To examine whether ISL can trigger autophagy in HEC-1A cells, we utilized TEM. TEM images revealed early autophagic bodies (autophagosomes) harboring intact organelles in HEC-1A cells after treatment with ISL for 12 h (Figure 5A). In addition, the autophagy markers LC3 and SQSTM1/p62 were measured after treatment with ISL. As shown in Figure 5B, ISL significantly increased the level of phosphatidylethanolamine-conjugated LC3-II and SQSTM1/p62 compared to that in control cells (Figure 5B).

To further determine the molecular mechanism underlying the activation of autophagy in HEC-1A cells, we measured the phosphorylation levels of ERK, a critical pathway for autophagy induction. As shown in Figure 5B, ISL treatment for 24 h concentration-dependently increased the expression of p-ERK, indicating that ISL activated ERK in HEC-1A cells. Pretreatment of HEC- 1A cells with the ERK inhibitor PD98059 (20 μM, 1 h) significantly blocked ISL-mediated activation of ERK as well as suppressed the up-regulation of LC3BII and cleaved caspase-7 (Figure 5C). These results suggest that the ERK signaling pathway is required for ISL-induced activation of apoptosis and autophagy.

To clarify the effect of ISL-induced autophagy in HEC-1A cells, we evaluated ISL-mediated survival and apoptosis in cells pretreated with the lysosome inhibitor bafilomycin A1 (BAF-1). As shown in Figure 5D, co- treatment of HEC-1A cells with ISL and BAF-1 (100 nM, 4 h) slightly increased ISL-mediated sub G1 phase cell cycle arrest and decreased cell viability from 70.1% (ISL only) to 55.1% (ISL + BAF-1) (Figure 5E). Western blot analysis indicated that co-treatment with BAF-1 (100 nM, 4 h) and ISL upregulated LC3-II and SQSTM1/p62 levels (Figure 5F) and enhanced the protein levels of cleaved caspase-7 and γH2AX in HEC-1A cells more than either treatment alone (Figure 5G). Consistent with the western blotting data, immunofluorescence analysis indicated that pretreatment with BAF-1 enhanced the fluorescence intensity of LC3 in ISL-treated HEC-1A cells (Figure 5H). Taken together, these data demonstrated that ISL triggers a protective autophagy in HEC-1A cells. Further investigation will be necessary to elucidate the relationship between autophagy and apoptosis after ISL treatment in HEC-1A cells.

### ISL suppresses the tumor growth *in vivo*

**Figure 6:**
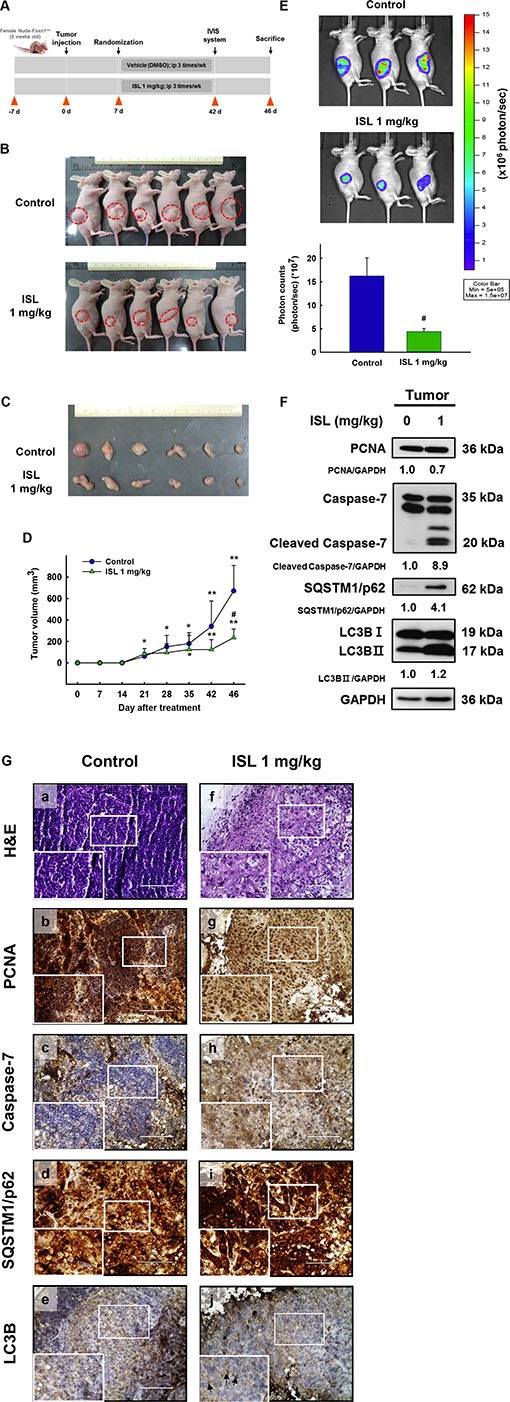
Effect of ISL treatment on the HEC-1A-LUC mouse xenograft model Tumors were implanted into mice by subcutaneous injection of HEC-1A-LUC cells (1 × 10^6^ cells/100 μl phosphate buffered saline (PBS) on the flank. After the tumors reached 50–100 mm^3^ in size, the mice received every 3-4 days an intraperitoneal injection of ISL (1 mg/kg) or vehicle (dimethylsulfoxide (DMSO)) for 1.5 months. (**A**) Schematic representation of the treatment plan. (**B**) Morphology and (**C**) size of the tumors of each group isolated from the sacrificed mice at day 46 (the end point) of treatment. (**D**) Tumor volumes of nude mice. (**E**) Total flux from luciferase imaging on day 42 after heterotopic tumor cell injection. All luciferase images were normalized to the same photon saturation scale. (**F**) Western blot analysis showed that ISL treatment reduced PCNA levels and increased caspase-7, SQSTM1/p62, and light chain 3B (LC3B) level in whole cell extracts from mouse tissues. (**G**) Tumors were excised and processed for hematoxylin and eosin (H&E) staining and immunohistochemical analysis of proliferating cell nuclear antigen (PCNA), caspase-7, SQSTM1/p62, and LC3B. Original magnification, ×200. Data are presented as the mean ± SD (*n* = 6). *p < 0.05 and **p < 0.001 vs. day 0; ^#^*p* < 0.05 vs. control.

Based on the above *in vitro* findings, we next conducted *in vivo* experiments to confirm the effect of ISL on xenograft animal models. As shown in Figure 6A, tumors were implanted into the athymic nude mice by subcutaneous injection of HEC-1A-LUC cells (1 × 10^6^ cells/100 μl PBS) into the flank. After the tumors reached 50–100 mm^3^ in size, the mice were randomly divided into two groups and received an intraperitoneal injection of ISL (1 mg/kg) or vehicle (DMSO) every 3–4 days for 46 days. At the end of the experiment, the tumor size of the ISL group was found to be significantly smaller than that of the vehicle group (Figure 6B and 6C). Tumor volume was monitored, and tumor growth in the ISL group was dramatically retarded compared with that of the vehicle group (Figure 6D). Total flux from luciferase imaging was statistically different on day 42 after heterotopic tumor cell injection. Bioluminescence analysis revealed that tumor growth was inhibited in mice treated with ISL 1 mg/kg compared to that in control mice (Figure 6E). To clarify the mechanism underlying ISL-induced suppression of xenograft tumor growth, the protein expression of PCNA, caspase-7, SQSTM1/p62, and LC3B was detected by western blot. ISL treatment significantly increased the expression of these proteins compared with the vehicle group (Figure 6F). Similarly, immunohistochemical analysis revealed that ISL inhibited the proliferation of cancer cells and decreased nuclear staining with H&E compared with the results observed for the vehicle group (Figure 6G. a–b). In addition, ISL treatment decreased the expression of PCNA (Figure 6G. c–d) and induced the expression of caspase-7, SQSTM1/p62, and LC3B compared with the vehicle group (Figure 6G. e–j). Taken together, these data demonstrate that ISL can suppress the growth of xenograft tumors *in vivo*.

## DISCUSSION

ISL has been reported to have anti-carcinogenic effects in both *in vivo* and *in vitro* experimental models [19–25]. In the present study, ISL showed an anti-proliferation effect on endometrial cancer cells. We found that ISL inhibited proliferation and cell cycle arrest in G1 or G2/M phase, promoted apoptosis and autophagy, and suppressed the growth of xenograft tumors *in vivo*. To the best of our knowledge, this is the first study to demonstrate directly ISL-induced suppression of human endometrial cancer growth *in vivo*.

Kim et al. investigated the effect of ISL on normal endometrial cells (human normal endometrial cell line T-HESCS) and found that 20 μM ISL was not toxic to these cells and that 50 μM ISL decreased survival to 76% [26]. Similarly, we found that ISL was not toxic to normal endometrial cells. Cells in these experiments were treated with 5–100 μΜ of ISL. Using the MTS assay, we found that doses of ISL up to 25 μΜ significantly suppressed endometrial cancer cell growth and reduced survival rate by approximately 50%. Similarly, cell counting with trypan blue staining revealed that treatment with ISL 25 μΜ for 48 h significantly reduced the ratio of dead to total number of cells.

ISL has been reported to inhibit the growth of different cancer cells by inducing cell cycle arrest, e.g., inducing G2/M phase arrest in oral squamous cell carcinoma cells and inducing G1 or G2/M phase arrest in prostate cancer cells [27–28]. Accumulating evidence shows that γH2AX is required for cell cycle arrest via the p53/p21 pathway [29]. In the repair process of damaged DNA, another key factor is γH2AX, which is recruited to damage sites [30]. Hsu et al. demonstrated that ISL induced Hep G2 cell apoptosis via the p53-dependent pathway in Hep G2 cells and that the upregulation of p53 induced p21/WAF1 expression and caused cell cycle arrest [31]. Similarly, ISL exposure increased the expression of γH2AX, p53, and p21 Cip in Ishikawa and HEC-1A cells.

In addition to promoting cell cycle arrest, ISL is known to induce apoptosis in a variety of human cancer cells. Hirchaud et al. found that ISL induced Ca Ski cell apoptosis by increasing caspase activities and inducing cleavage of caspase-3 and its downstream substrate PARP [32]. In addition, Chen et al. demonstrated that both the ratio of Bax/Bcl-2 and the cleavage of PARP significantly increased in ISL-treated antibody-containing cells (ACC) cells [33]. In our study, we found that treatment with 50 μΜ ISL significantly induced apoptosis by upregulating the cleavage of caspase-3 and −7 and PARP expression in Ishikawa or HEC-1A cells. These data suggest that apoptosis plays a key role in cancer cell death. However, more studies are needed to clarify the role of the apoptotic factors in endometrial cancer cells.

Natural compound-induced autophagy may be either pro-survival or pro-death in cancer therapy. LC3 is a specific autophagy marker that is recruited to the autophagosomal membrane. During autophagy, LC3-I is conjugated to autophagic membrane-associated phosphatidylethanolamine (PE) and converted to LC3-II. Increased LC3-II expression, especially an increase in the LC3-II/LC3-I ratio, may indicate the occurrence of autophagy. Therefore, we measured levels of LC3II as an indicator of the autophagy in our studies. In general, inhibition of autophagy is correlated with increased levels of p62, suggesting that steady-state levels of this protein reflect autophagic status [34]. Our results indicate that p62 accumulates in ISL-treated cells and that the breakdown of autophagolysosomes must be inhibited by ISL. These results are similar to those of a previous study, which showed that capsaicin treatment induced an increase in cytoplasmic LC3 puncta that co-localizes with lysosomes. The authors suggested that the formation of autophagolysosomes was greater than its breakdown, promoting its accumulation in the cell [35]. In our study, we found that ISL significantly increased the expression of LC3II and elevated the levels of p-ERK in a dose-dependent manner. These findings are similar to previous studies that found that ERK-dependent autophagic activity was associated with induction of LC3 and the conversion of LC3I to LC3II [36–37].

Autophagy inhibitors are classified as early- or late-stage inhibitors of the pathway. Early-stage inhibitors include 3-methyadenine, wortmannin, and LY294002, whereas late-stage inhibitors include the anti-malarial drugs CQ, hydroxychloroquine (HCQ), BAF-1, and monensin [38]. BAF-1 is a specific inhibitor of vacuolar-ATPase. Treatment with BAF-1 results in elevated lysosomal pH and blockade of the fusion of the autophagosome with the lysosome [39], thereby achieving suppression of the downstream autophagy [40]. In our study, we found that ISL significantly increased the expression of LC3-II and cleaved caspase-7 following BAF-1 treatment. Furthermore, combination treatment of ISL with BAF-1 significantly decreased the cell viability of HEC-1A. These results are similar to those of a previous study, which found that inhibition of autophagy in malignant glioma cell lines by treatment with BAF-1 and arsenic trioxide led to cell death by apoptosis instead [41].

The *in vivo* experiments verified our *in vitro* findings on the effects of ISL against human endometrial cancer. Tumor size, but not body weight, significantly decreased after administration of 1 mg/kg of ISL intraperitoneally, suggesting that 1 mg/kg ISL did not cause side effects in mice. In a previous study, Hsia et al. fed mice more than 1 mg/kg of ISL and found that the average tumor size also decreased [27]. Using a xenograft animal model to investigate the effects of ISL on the tumor growth of NCI-H1975 cells, Jung et al. found that intraperitoneal administration of 1 or 5 mg/kg of ISL dramatically retarded tumor volume in ISL groups compared with the vehicle-treated group [42]. These findings suggest that ISL is a potential anti-cancer drug candidate.

In a previous pharmacokinetic-related study, the plasma concentration of ISL 30 min after administration of a 50 mg/kg intravenous dose of ISL to rats was 4.16 ± 1.80 mg/mL. However, the plasma ISL concentration within 30 min of an oral dose of 100 mg/kg was 0.604 ± 0.689 mg/mL. Therefore, intravenous administration of ISL was more effective than oral administration [11]. Regardless of the delivery method of ISL, the absorption rate and discharge rate are rapid [43].

**Figure 7:**
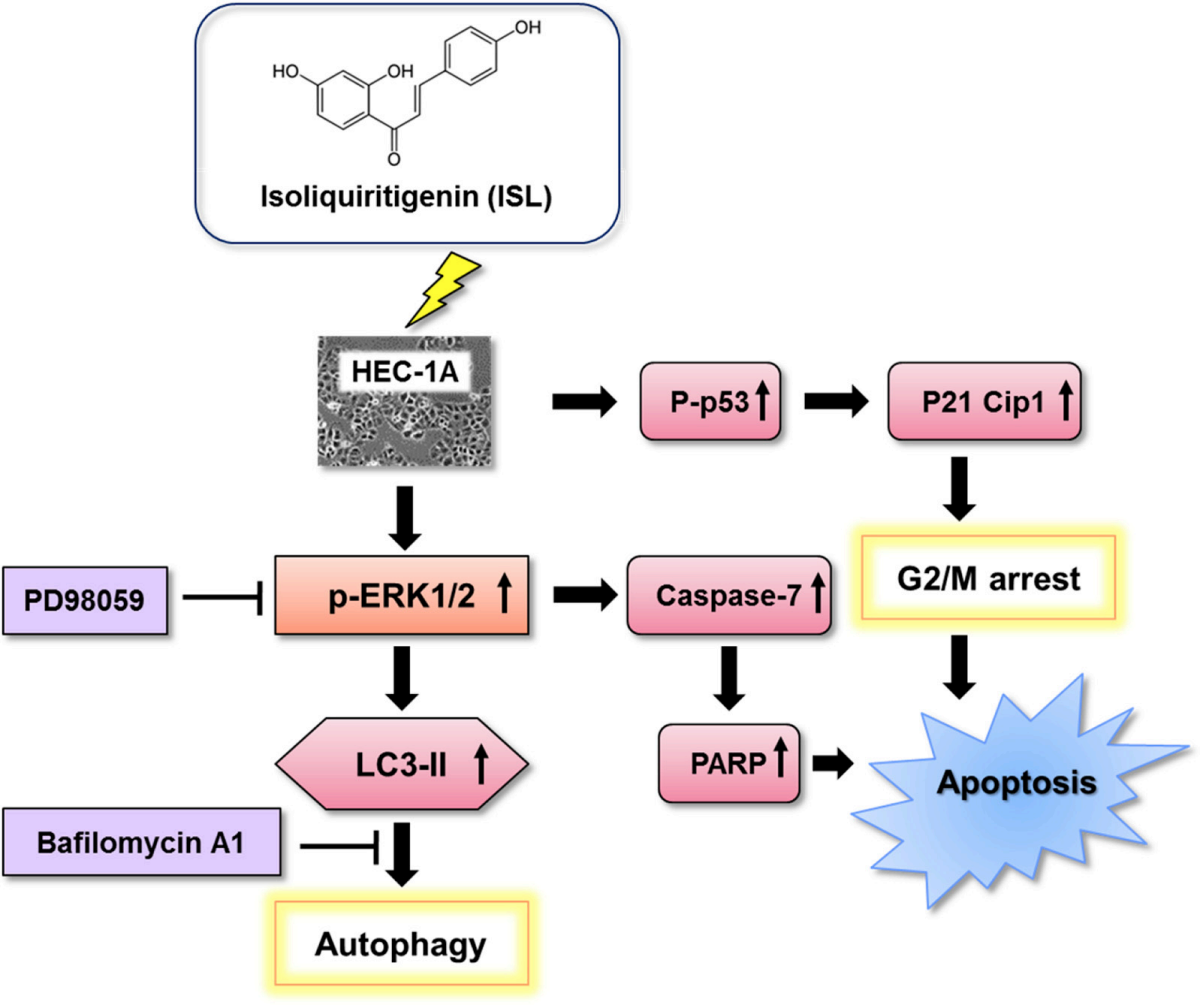
Schematic diagram of the mechanism of ISL-induced programmed cell death

In conclusion, the present study demonstrated that ISL induced cell cycle arrest in the sub-G1 or G2/M phase of endometrial cancer cells via the p53/p21 signaling pathway and that ISL treatment suppressed HEC-1A-LUC xenograft tumor growth by inhibiting PCNA expression and increasing caspase-7, p62, and LC3B expression in tumor tissues. Moreover, ISL increased the levels of ERK, LC3B-II, caspase-3, caspase-7, and PARP in endometrial cancer cells, which suggests that ISL activated the ERK signaling pathway to induce both apoptosis and autophagy in HEC-1A cells. Thus, ISL may be an effective candidate agent for the treatment of human endometrial cancer (Figure 7).

## MATERIALS AND METHODS

### Reagents and antibodies

McCoy's 5A medium, sodium bicarbonate, dimethyl sulfoxide (DMSO), DNAse-free RNAse A, propidium iodide (PI), and Triton X-100 were purchased from Sigma-Aldrich (St. Louis, MO, USA). Minimum essential medium (MEM), Dulbecco's Modified Eagle's Medium (DMEM)/Ham's F-12 1:1 mixture medium, antibiotic-antimycotic solution (100X), and 0.05% trypsin-EDTA (1X) were purchased from Caisson Labs (Smithfield, UT, USA). Fetal bovine serum (FBS), trypan blue, bicinchoninic acid (BCA) protein assay kit, and electrochemiluminescence (ECL) immunoassay were purchased from Thermo Fisher Scientific (Boston, MA, USA). Bovine serum albumin (BSA) was purchased from BioShop (Burlington, Canada). A protease inhibitor cocktail tablet and phosphatase inhibitor cocktail tablet were purchased from Roche (Basel, Switzerland). Annexin V-fluorescein isothiocyanate (FITC) Apoptosis Detection Kit I was purchased from Becton Dickinson (BD) Biosciences (San Diego, CA, USA). CellTiter 96^®^ AQueous One Solution Cell Proliferation Assay (MTS assay) and VivoGlo™ Luciferin (*In Vivo* Grade) were purchased from Promega (Madison, WI, USA). The following antibodies were used in this study: anti-light chain-3B (LC-3B) from Novus Biologicals (Littleton, CO, USA); anti-SQSTM1/p62 and anti-p21 Cip1 from GeneTex (Irvine, CA, USA); anti-caspase-3, anti-caspase-7, anti-cleaved-poly adenosine diphosphate-ribose polymerase (PARP), anti-p-extracellular signal regulated protein kinase (ERK)1/2, anti-ERK1/2, anti-p-γH2AX, anti-proliferating cell nuclear antigen (PCNA), and anti-p-p53 (Ser15) from Cell Signal Technology (Beverley, MA, USA); anti-horseradish peroxidase (HRP)-conjugated glyceraldehyde 3-phosphate dehydrogenase (GAPDH) from Proteintech (Rosemont, IL, USA); and goat anti-rabbit/mouse antibody IgG from Abcam (Cambridge, UK). Bafilomycin A1 (BAF-1) was purchased from Invitrogen Life Technologies (Carlsbad, CA, USA).

### Preparation of ISL

ISL (C_15_H_12_O_4_; CAS number: 961–29–5), a yellow needle-shaped crystal powder that is insoluble in water was purchased from Sigma-Aldrich with purity over 98%. A stock solution of 100 mM was prepared in DMSO and aliquoted and stored at −20°C until use. For all experiments, the final concentrations of the test compound were prepared by diluting the stock with medium. The carrier solvent (0.1% DMSO) was added to the control cells.

### Cell culture

Human endometrial cancer cell lines (HEC-1A and RL95-2) were purchased from Food Industry Research and Development Institute (FIRDI, Taiwan, ROC) and Culture Collection and Research Center (CCRC, Taiwan, ROC). Human endometrial adenocarcinoma cell line (Ishikawa) was purchased from the European Collection of Authenticated Cell Culture (ECACC, Salisbury, UK). Human endometrial stromal cell lines (telomerase-immortalized human endometrial stromal cells (T-HESCs) were purchased from ATCC (American Type Culture Collection, Manassas, VA, USA). HEC-1A cell line was cultured in McCoy's 5A. RL95-2 cell line and T-HESCs were cultured in DMEM/Ham's F-12. Ishikawa cell line was cultured in MEM. These cells were maintained in media containing 10% FBS (Ishikawa cells were grown in medium supplemented with 5% FBS) and 1% antibiotics (10,000 units/mL penicillin, 10,000 μg/mL streptomycin, 25 μg/mL amphotericin with 8.5 g/L NaCI) and incubated at 37°C with 5% CO_2_.

### Cell viability assay

The effect of ISL treatment on cell viability was examined using the MTS assay. Cells were seeded in 96-well plates (3 × 10^3^ cells per well), cultured for 24 h, and then treated with various concentrations of ISL in fresh medium containing 1% serum. The reagent mixture (MTS/PMS) was added directly to each well at 24 and 48 h at the recommended ratio of 20 μl of reagent to 100 μl of culture medium. Absorbance was measured at 490 nm with reference wavelength of > 630 nm using an enzyme linked immunosorbent assay (ELISA) reader (BioTek, Winooski, VT, USA).

### Trypan blue exclusion test

After cell adhesion, the medium was replaced with fresh medium containing 1% serum and various concentrations of ISL. After 48 h, the cells were harvested in culture medium with 0.05% trypsin-EDTA solution from cell culture dishes. Cells and medium were collected at 4°C, centrifuged at 500 *g* for 5 min, and resuspended in 1 ml medium mixture. Ten microliters was removed, and trypan blue staining was used to determine the number of dead cells. Using a hemocytometer, the total number of live and dead cells on the counting disk (cell counter) on four squares (top left, top right, bottom right, lower left) were determined, and the ratio of dead to total number of cells was calculated.

### Cell cycle analysis

To assess cell cycle progression, cells were seeded onto culture dishes, incubated for 24 h to allow for exponential growth, and treated with ISL at the indicated concentrations for 48 h. All cells were collected, and after addition of 9 ml of 70% ethanol, they were stored at −20°C for at least 2 h. Cells were washed at least once with cold phosphate buffered saline (PBS) and then resuspended in 300–500 μl propidium iodide (PI)/Triton X-100 staining solution (to 10 ml of 0.1% (v/v) Triton X-100 in PBS, 2 mg DNAse-free RNAse A and 0.40 ml of 500 μg/ml PI were added) and incubated for 30 min at 20°C. Fluorescence was measured with a FACSCalibur flow cytometer (BD, San Jose, CA, USA), and cell cycle distribution was analyzed using CellQuest and Modfit LT programs (BD).

### Apoptosis analysis

For the apoptosis analysis, Annexin V-FITC Apoptosis Detection Kit I was used. The cells were seeded in culture dishes, were treated with ISL at the indicated concentrations for 48 h, and stained with Annexin V-FITC and PI. The cells were incubated for 15 min at room temperature and protected from light. Apoptosis was analyzed using FACSCalibur flow cytometer, and the results were analyzed using the CellQuest software program.

### Western blot analysis

Endometrial cancer cell line lysates were prepared in ice-cold lysis buffer (50 mmol/l Tris (pH 8.0), 100 mmol/l NaCl, 0.1 % sodium dodecyl sulfate (SDS), 1% nonidet P-40 (NP-40), 0.5 mM EDTA) containing protease inhibitor and/or complete phosphatase inhibitor cocktail. Proteins (20 μg) were boiled for 5 min and then subjected to 15 or 10% SDS-polyacrylamide gel electrophoresis (PAGE). The proteins were transferred electrophoretically to Immobilon-P Membrane (polyvinylidene fluoride (PVDF) membrane, 0.45 μm) for 150–180 min at 280 mA and 250 V. Membranes were washed three times for 10 min each with Tris buffered saline (TBS) with Tween 20 (TBST). The membrane was blocked with blocking buffer (5% BSA) for 1 h at room temperature and reacted overnight with the immunoaffinity-purified antibodies (Supplementary Table S1). The next day, membranes were washed three times for 10 min each with TBST, incubated for 1 h in blocking buffer with anti-rabbit IgG (1:6,000) coupled to alkaline phosphatase, and washed three times for 10 min each with TBST buffer. Proteins were detected using ECL, and protein values were normalized to an internal control, GAPDH. Densitometric estimations were quantified using Image J software (National Institutes of Health, Bethesda, MD, USA).

### Immunofluorescence analysis

Cells were cultured on sterile glass coverslips and treated with ISL (25 μM, 24 h) after treatment with BAF-1 (100 nM, 4 h). Cells were then fixed with 4% formaldehyde at room temperature for 15 min. The cells were rinsed twice with 1X PBS, permeabilized using 0.1% triton X-100/PBS for 15 min, and blocked in blocking solution (5% (w/v) BSA in 1X TBST). Cells were incubated with primary antibodies (Supplementary Table 1) overnight at 4°C. Cells were rinsed thrice in 1X PBS for 15 min and then incubated with secondary antibodies (Alexa Fluor^®^ 488 dye, LifeTechnologies, Gaithersburg, MD, USA) for 1 h at room temperature and then rinsed four times in 1X TBST for 20 min. ProLong^®^ Gold Antifade Mountant was added to the cells. Fluorescent images were taken using an EVOS^®^ microscope (Thermo Fisher Scientific).

### Immunohistochemistry

Tissue slides were incubated at 65°C for 30 min and then subjected to deparaffinization in xylene for 10 min in a microwave oven for antigen retrieval. After washing with 1·NaCl/Pi, the slides were immersed in 3% hydrogen peroxide for 10 min to suppress endogenous peroxidase activity. After triple rinses with 1·NaCl/Pi, sections were incubated with primary antibodies (Supplementary Table S1) for 1 h at room temperature. After triple rinses with 1·NaCl/Pi, the slides were incubated with biotinylated secondary antibody (Dako, Glostrup, Denmark) for 25 min. Following triple rinses with 1 NaCl/Pi, horseradish peroxidase-conjugated streptavidin was added for 25 min at room temperature. The peroxidase activity was detected with alkaline earth cuprate (AEC+) substrate chromogen (Dako) at room temperature. The slides were then counterstained with hematoxylin. Images were taken using an EVOS^®^ microscope.

### Transmission electron microscopy (TEM)

Cells were fixed with 4% paraformaldehyde and 0.1% glutaraldehyde in 0.1 M phosphate buffer, pH 7.4, and postfixed with 1% OsO4 for 2 h. The cells were then dehydrated with increasing concentrations of alcohol (30, 50, 70, 90, and 100%), infiltrated with LR White resin two times for 1 h each, and subsequently embedded in LR White resin. The solidified blocks were cut into 60 nm thicknesses and were stained with uranyl acetate and lead citrate. Samples were observed under a transmission electron microscope (Hitach HT-7700; Hitachi, Tokyo, Japan).

### Tumor xenograft in nude mice

Five-week-old female Nude-Foxn1^nu^ mice (BioLASCO, Taipei, Taiwan) were housed under a 12 h light/12 h dark cycle in a pathogen-free environment, with food and water available *ad libitum*. Tumors were implanted by subcutaneous injection of HEC-1A-LUC cells (1 × 10^6^ suspended in 0.1 mL PBS for each mouse) into the flank of mice. After the tumors reached 50–100 mm^3^ in size, mice were randomly divided into two group (*n* = 6) and received every 3–4 days an intraperitoneal injection of ISL (1 mg/kg) or vehicle (DMSO) for 46 days. Tumor volume was measured with calipers and was calculated as L × W^2^ × 0.52, where L is the length and W is the width. Tumor volumes and body weight were recorded until animals were sacrificed. Every week, the mice were injected intraperitoneally with 150 mg/kg of body weight luciferin and detected using an *in vivo* imaging system (IVIS). At the end of the experiment, the tumors were formalin-fixed and stained with hematoxylin and eosin (H&E), PCNA, caspase-7, SQSTM1/p62, and LC3B. All animal studies were conducted according to the protocols approved by the Institutional Animal Care and Use Committee (IACUC) of Taipei Medical University (IACUC Approval No. 2015–0115).

## SUPPLEMENTARY MATERIALS TABLE


