# Supplementary material for: Isoliquiritigenin induces apoptosis and autophagy and inhibits endometrial cancer growth in mice
Source: Oncotarget. 2016 Sep 30;7(45):73432–47. doi: 10.18632/oncotarget.12369 (PMC5341989; doi:10.18632/oncotarget.12369)
Supplement: Supplementary file 1 [file oncotarget-07-73432-s001.pdf]

Isoliquiritigenin induces apoptosis and autophagy and inhibits endometrial cancer growth in mice

Supplementary Materials

Supplementary Table S1: Antibody used in this manuscript

Antibody name	No.	Ratio	Brand
LC3B	NB100-2220	1:1000	Novus Biologicals (CO, USA)
SQSTM1/p62	GTX629890	1:1000	GeneTex (CA, USA)
p21 Cip1	GTX629543	1:500	GeneTex (CA, USA)
LC3B	#3868	1:3200 (IHC)	Cell Signal Technology (MA, USA)
Caspase-3	#9662	1:1000	Cell Signal Technology (MA, USA)
Caspase-7	#9492	1:2000	Cell Signal Technology (MA, USA)
PARP	#9542	1:1000	Cell Signal Technology (MA, USA)
p-ERK1/2	#9101	1:1000	Cell Signal Technology (MA, USA)
ERK1/2	#9102	1:1000	Cell Signal Technology (MA, USA)
p-p53 (Ser15)	#9284	1:1000	Cell Signal Technology (MA, USA)
PCNA	#2586	1:1000 (WB)<br>1:4000 (IHC)	Cell Signal Technology (MA, USA)
HRP-conjugated GAPDH	HRP-60004	1:10000	Proteintech (IL, USA)
Goat anti-rabbit antibody IgG	ab6721	1:10000	Abcam (MA, USA)
Goat anti-mouse antibody IgG	ab6789	1:10000	Abcam (MA, USA)
Caspase-7	ab69540	1:100 (IHC)	Abcam (MA, USA)
SQSTM1/p62	ab56416	1:1000 (IHC)	Abcam (MA, USA)
p-γH2AX (Ser139)	05-636	1:1000	EMD Millipore (MA, USA)
